# Hyaluronan nanoscale clustering and Hyaluronan synthase 2 expression are linked to the invasion of child fibroblasts and infantile fibrosarcoma in vitro and in vivo

**DOI:** 10.1038/s41598-022-21952-4

**Published:** 2022-11-18

**Authors:** Joseph J. Tonge, Scott V. Notley, Mark J. Dunning, Ana López-Guajardo, Jessica D. Medcalf, Paraskevi Heldin, George Panoutsos, Annica K. B. Gad

**Affiliations:** 1 Academic Unit of Medical Education, The Medical School, The University of Sheffield, Sheffield, UK; 2 Department of Oncology and Metabolism, The Medical School, The University of Sheffield, Sheffield, UK; 3 Department of Automatic Control and Systems Engineering, The University of Sheffield, Sheffield, UK; 4 Bioinformatics Core, The Medical School, The University of Sheffield, Sheffield, UK; 5 Department of Medical Biochemistry and Microbiology, Uppsala University, Uppsala, Sweden; 6 Centro de Química da Madeira, Universidade da Madeira, Funchal, Portugal

**Keywords:** Cancer, Cell biology, Cancer

## Abstract

Infantile fibrosarcoma is a rare childhood tumour that originates in the fibrous connective tissue of the long bones for which there is an urgent need to identify novel therapeutic targets. This study aims to clarify the role of the extracellular matrix component hyaluronan in the invasion of child fibroblasts and Infantile fibrosarcoma into the surrounding environment. Using nanoscale super-resolution STED (Stimulated emission depletion) microscopy followed by computational image analysis, we observed, for the first time, that invasive child fibroblasts showed increased nanoscale clustering of hyaluronan at the cell periphery, as compared to control cells. Hyaluronan was not observed within focal adhesions. Bioinformatic analyses further revealed that the increased nanoscale hyaluronan clustering was accompanied by increased gene expression of Hyaluronan synthase 2, reduced expression of Hyaluronidase 2 and CD44, and no change of Hyaluronan synthase 1 and Hyaluronidases 1, 3, 4 or 5. We further observed that the expression of the Hyaluronan synthase 1, 2 and 3, and the Hyaluronidase 3 and 5 genes was linked to reduced life expectancy of fibrosarcoma patients. The invasive front of infantile fibrosarcoma tumours further showed increased levels of hyaluronan, as compared to the tumour centre. Taken together, our findings are consistent with the possibility that while Hyaluronan synthase 2 increases the levels, the Hyaluronidases 3 and 5 reduce the weight of hyaluronan, resulting in the nanoscale clustering of hyaluronan at the leading edge of cells, cell invasion and the spread of Infantile fibrosarcoma.

## Introduction

Hyaluronan, also referred to as hyaluronic acid, is a glycosaminoglycan chain molecule that is required for cell motility and cell migration^1^. Hyaluronan is present in most extracellular matrices within normal and cancer tissues^2^, and the deposition of hyaluronan is significantly increased in various cancers^3,4^. Hyaluronan differs from other glycosaminoglycan. First, it can be much larger with a molecular weight ranging between 5 and 20,000 kDa, secondly, in contrast to all other glycosaminoglycan which are synthesised at Golgi, hyaluronan is synthesised at the plasma membrane and directly secreted into the extracellular matrix^1^. Hyaluronan is synthesised by the Hyaluronan synthases 1–3, of which Hyaluronan synthase 1 and 2 produce higher molecular weight chains than Hyaluronan synthase 3 (~ 2 × 10^6^ Da *versus* ~ 2 × 10^5^ Da), respectively^1^. Hyaluronan is found mostly as high molecular variants, which inhibits cell motility and tumour growth^1,5,6^. These long linear chains of hyaluronan can be digested to hyaluronan fragments of low molecular weight by hyaluronidases. In contrast to the high molecular weight variants, low molecular weight hyaluronan promotes local metastatic spread of primary tumours, as highlighted by the observation that an inactivating point mutation in the hyaluronan-degrading enzyme Hyaluronidase 3 suppresses the growth of tumours in naked mole-rats^7^. While the Hyaluronidases 2 and 3 exclusively degrade hyaluronan, the Hyaluronidases 1 and 5 (also called PH20/SPAM1) also degrade Chondroitin Sulfate^3^. The Hyaluronidase 4 has a similar protein structure as hyaluronidases, but no hyaluronidase activity^3^. However, how hyaluronan regulates cell adhesion and why lower and higher weight chains of hyaluronan have opposite effects on cell motility remain to a large degree unknown.

The binding of the cell to hyaluronan is considered one of the initial events in the formation of cell-extracellular matrix, and it can potentiate integrin signalling^8^. Binding of extracellular hyaluronan to the membrane receptors CD44 and RHAMM promotes cell motility and invasion^6^, and targeting of hyaluronan and downstream signalling has potential for future cancer therapy^9^. In particular, in fibrosarcoma cells, the low, but not high molecular variants of hyaluronan result increase cell adhesion to the extracellular matrix, via a mechanism that requires RHAMM but not CD44^6^.

Infantile fibrosarcomas are rare childhood tumours that usually originate in the fibrous connective tissue of the long bones. Histologically, they show increased nuclear mitotic activity with immature spindle fibres. The primary treatment is invasive surgery to resect the tumour. This often leads to prolonged stays in hospital, bleeding, and complications associated with complex surgery^2,10^, and there is an urgent need to identify drugs that can be used as an adjuvant therapy to reduce spread and the size of the tumour before surgery. It is known that invasive and metastasizing tumour cells have reduced capacity to form cell–matrix adhesions and adhere^11–13^. We therefore hypothesised that hyaluronan induces the motility and invasion of fibroblast cells within Infantile fibrosarcomas. Knowledge of how hyaluronan is distributed in tissues and cells, and how the gene expression of hyaluronan-related proteins change during the metastatic transformation of child fibroblasts can identify novel therapeutic targets for the development of future strategies that suppress the spread of the disease, allowing less complex and invasive surgery^10^. In the present study, we therefore aimed to identify if changes in the spatial distribution of hyaluronan, both at the tissue and subcellular, nanoscale level, and the gene expression of hyaluronan synthases and hyaluronidases that accompany the spread of child fibrosarcoma.

## Materials and methods

### Cell types and Infantile fibrosarcoma tissue

We used primary human newborn fibroblasts immortalised with the catalytic domain of telomerase as normal control cells (Bjhtert), and the isogenetically matched, transformed, invasive, and metastasising counterpart of these cells, BjhtertSV40THRasV12 as invasive cells (Bj-invasive)^14,15^. These cells were created by, and a kind gift from Hahn et al.^14^. However, the Bjhtert cells are commercially available at ATCC (CRL-4001). To our knowledge, the BjhtertSV40THRasV12 cells are not commercially available. The BJ-cell model system was selected because it is, to our knowledge, the only model that allow a direct comparison between isogenically matched healthy and invasive child fibroblasts. We obtained fresh tumour sections with central and peripheral regions from patients with infantile fibrosarcoma from the Paediatric oncology team, at the Sheffield Children’s Hospital, Sheffield, UK.

### Immunofluorescent staining

We seeded 300,000 cells in 6-well plates with circular glass coverslips (20 mm). After 43 h, cells were fixed and stained for hyaluronan, and F-actin as follows. After a 2 min wash in Dubelcco’s modified Basal Eagle Medium with no supplements, cells were fixed for 15 min RT in 3.7% Formaldehyde with 1% Methanol in phosphate-buffered saline (PBS), followed by a 3 min 0.2% Triton X100 permeabilization step, and a 5 min wash in PBS. Fluorescence was then quenched with 30 min RT incubation in 100 mM Glycine. After two quick, 30 s, washes in PBS/10% EtOH, in which EtOH was included to avoid losing cell-associated hyaluronan, the samples were blocked for 15 min RT with streptavidin, followed by 15 min RT biotin-treatment, and 30 min RT in 3% (w/v) bovine serum albumin (BSA)/10% goat serum in PBS. After two quick washes in PBS/10% EtOH, samples were incubated overnight at 4 °C with 4 µg/ml Biotin-HABP (Merck Life Science UK Limited, Germany) in PBS with 1% (w/v) BSA. As a negative control, one coverglass was incubated for 1–5 h RT with 4 µg/ml Biotin-HABP with 100 µg/ml HMW-hyaluronan in PBS with 1% PBSA, followed by a 1 h RT incubation with 1.5 µg/ml Streptavidin-Abberior STAR635P (Abberior GmbH, Germany) in PBS with 1%BSA, a brief wash, and a 1 h RT incubation with the 4.5 µg/ml of the secondary anti-mouse-Alexa594 antibody and 13.2 nM Phalloidin-OregonGreen/488/Green (Thermo Fisher Scientific, USA) in PBS with 1% BSA. After one quick, one 10 min, and one 45 min wash in PBS/10% EtOH, glasses were dipped in distilled water, gently dried and mounted in Mowiol 18 (Merck Life Science Limited, UK).

### Immunohistochemistry

We dewaxed 9 paraffin-embedded, 5 µm transverse sections of whole Infantile fibrosarcoma tumours from one patient, and blocked the samples in 1% (w/v) BSA in PBS for 30 min RT. This was followed by a second blocking step using the Endogenous Biotin-Blocking kit (Invitrogen, ThermoFisher Scientific UK) according to the manufacturer’s instructions, followed by an overnight incubation at 4 °C with 2.5 µg/ml b-HABP in 1% (w/v) BSA in PBS as described earlier^16^. This was followed by a 10 min RT wash in PBS with 0.1% Tween-20, 30 min RT incubation in 1:1000 Streptavidin-Alexa 594 conjugate (Invitrogen, ThermoFisher Scientific UK), after which the samples were mounted in Vectashield Antifade Mounting Medium with DAPI (Vector Laboratories, US). Thereafter, all samples were captured with identical settings and exposure times, at 488 nm and 594 nm on an Olympus FV1000 BX61 confocal system using the FV10-ASW (version 4.2, https://bit.ly/3S8woiD) acquisition software (Olympus Corporation, Japan). Infantile fibrosarcoma tumours are very rare. To follow the local and national ethical guidelines that ensure that patients are not identified, the clinical features could therefore not be included in the manuscript.

### Microscopy

For Confocal microscopy, we used a Zeiss Axioplan 2 immunofluorescence microscope (Carl Zeiss AB, Sweden), the software Velocity, and NIS-Elements AR 3.2, 64-bit images. We captured the 594 and 488 signals with automatic exposure times, and the 635 signal with identical settings across all samples. For Stimulated Emission Depletion (STED) imaging, we used a Leica SP8 3X STED system equipped with lasers for depletion of fluorophores emitting in the blue/green (592 nm, MPB Communications Inc, Canada) and red/far-red (775 nm, NKT Photonics, Denmark). A chromatically optimised oil immersion objective (HC PL APO 100X/1.40 OIL STED WHITE, Leica Microsystems, Germany) was used for imaging and a tunable pulsed white-light fibre-laser emitting from 470 to 670 nm for excitation. Fluorescence signals were passed through a 0.85–0.9 Airy unit pinhole, dichroic mirrors optimised for each emission spectra, and STED laser notch filters placed in front of sensitive photodetectors (Leica Hybrid Detectors, Leica Microsystems, Germany). Triple-colour frames (1024 × 1024) were acquired sequentially at a scan speed of 100–200 lines per second with 3–4 line averages and a pixel size of 25 nm. Raw STED images were deconvoluted with the Huygens STED algorithm without any normalisation applied across all images^16^.

### Computational analysis of hyaluronan distribution

#### Image segmentation

Image segmentation was performed manually on each STED image, by expert mark-up of the outer region of each cell in or adjacent to the lamellipodia. Each image was divided into a grid, starting at the top left of the image, and with a constant block size of 2.52 × 2.52 microns (100 × 100 pixels). All grid blocks that were partially, or wholly, overlapped by the markup area were analysed (Suppl. Fig. S4).

#### Estimation of hyaluronan patch count, size and intensity

Analysis was performed on each grid block across all images to estimate the number and size of hyaluronan patches. Each grid block under analysis was binarised with a threshold determined by the Otsu’s method^17^. Each binarised block was analysed with a connected component analysis (eight connected) to find the number of connected components (corresponding to a hyaluronan patch) and the average number of pixels in each^18^. The binarized image was also used as a mask to find the average image intensity of the areas containing hyaluronan within each block.

#### Texture analysis

Each block, across all images, was analysed for texture by using a modified method of extracting Haralick features from the Gray Level Co-Occurrence Matrix (GLCM)^19^. GLCM based texture measures provide information related to the local spatial relationships of gray levels in an image and have previously been successfully applied in a number of medical imaging modalities^19–23^. One of the challenges in the standard application of the GLCM is the dependence on rotation and spatial offset, which is pertinent to this study. In this work we use the method proposed by Putzu and Di Ruberto to provide robust features invariant to rotation and spatial offset^24^. For each grid block, a GLCM matrix was formed using spatial offsets of: [dx,dy] = [0,1]; [0,2]; [0,3]; [0,4]; [1,0]; [2,0]; [3,0]; [4,0]; [1,1]; [2,2]; [3,3]; [4,4] accounting for 4 spatial offsets at 3 angles (0°, 45° and 90°). Haralick features for Energy, Contrast and Homogeneity were then derived for each GLCM matrix. The method was applied to each feature with the first eigenvalue used as the invariant form. Energy is a measure of local uniformity of the gray levels, contrast gives more weight to pixel combinations with large local variations and homogeneity measures how close the GLCM matrix is to diagonal; heterogeneity was defined as (1-homogeneity). For a more detailed description, please see the Supplementary Equations, in the Supplementary Information.

### RNA sequence analysis

Raw mRNA sequencing reads were obtained from the sequencing read archive (accession code SRP131149) and processed using the bcbio workflow (https://bcbio-nextgen.readthedocs.io/en/latest/)^25^. The transcripts from the human transcriptome (version GRCh37) were quantified using the salmon tool (version 0.9.1), followed by analysis in R (version 4.0.1) using the tximport package (version 1.161), and DESeq2 (version 1.28.1)^26,27^, to analyse differential expression, generate p-values and statistics for downstream analysis via a generalised linear model^28^.

### Literature review

A systematic literature review was carried out using Pubmed (National Center for Biotechnology Information, Rockville Pike, USA) and Medline Ovid (https://ovidsp.ovid.com, (accession date 11 September 2021), using the following combined search terms: “Hyaluronan”, “Fibroblast”, “Paediatric” and “Fibrosarcoma”. The inclusion criteria were articles focussing on child fibrosarcoma tissues, on cells expressing increased levels of Hyaluronan, or on cells producing short variants of Hyaluronan, and published in the English language 2000–2020. The exclusion criteria were articles not including Hyaluronan, fibrosarcoma, fibroblasts or children, and review articles (Suppl. Fig. S1).

### STRING analysis

A STRING protein interaction analysis was undertaken using the proteins identified in the literature review (https://string-db.org, String consortium, Lausanne, Switzerland). No limitations were applied in the analysis, and interactors of each protein were searched for in Homo sapiens and Mus musculus. Thereafter, we applied the limitations to include only experimental data and curated database data. The gene ensembl ID was provided by online database Genecard (https://www.genecards.org). We assessed the validity of the experimental data and if it, according to literature, had a possible role in cell motility.

### Cancer Genome Atlas analysis

To identify Hyaluronan-related genes that have a role in the progression of fibrosarcoma, we analysed the prognosis of patients in which the genes encoding for Hyaluronan synthase 1,2,3 or Hyaluronidase 1,2,3,4 or 5 was expressed in fibrosarcoma tumours. For this, we first compared the expression of the genes in fibrosarcoma of the biopsy histoimmunochemistry data, using the Cancer Genome Atlas online database (National Institutes of Health, Maryland, USA). Here, we searched for the gene of interest, in combination with the terms or phrases “Soft tissue tumours” and “Sarcoma”. All samples were ethically approved to be analysed and obtained from biotype protein coding^29^. Secondly, we visually analysed all soft tissue sarcomas samples obtained in this search, selecting the samples with a fibrosarcomatous histological appearance, and included only samples from children with sarcomas that were histologically determined to be similar to fibrosarcoma tumours in our study.

### Statistical analysis

The differences between the features derived from the computational image analysis were determined by the Mann–Whitney U tests^30^, as non-parametric tests of the null hypothesis of equal distributions. Since Mann–Whitney U tests are only tests of equality of medians under restricted conditions^31^, the medians were further assessed for equality, via bootstrap resampling, of the difference of medians between groups (Suppl. Fig. S6 and Suppl. Table S2). The graphs show notched box-plots with the notches representing 2 times the standard error of the median^32^.

### Ethics approval

HRA (Health Research Authority) and NHS (National Health Service) ethical approval was granted (reference 21/HRA/0744). Local approval was gained from Sheffield Children’s Hospital NHS Foundation Trust (reference SCH-2544). This study was conducted in accordance with the good ethical guidelines of the University of Sheffield (reference 035785).

### Consent to participate

Informed consent was obtained from all participants or, if participants are under 18, from a parent and/or legal guardian.

## Results

### Transformed and invasive child fibroblasts show increased hyaluronan at the cell periphery

**Figure 1:**
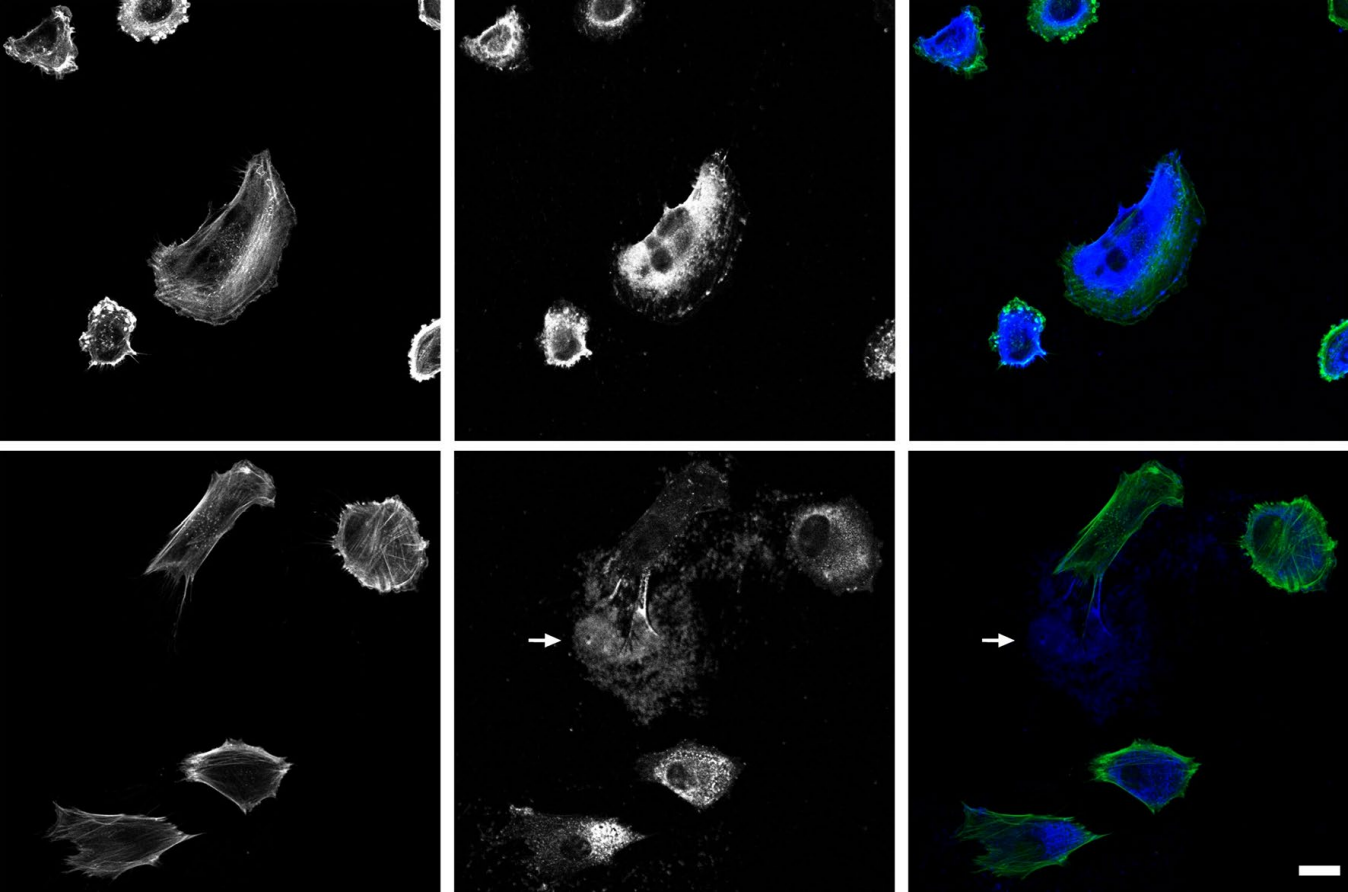
Spatial distribution of hyaluronan and F-actin in control and invasive fibroblasts. Images show normal Bjhtert (bottom) and Bj-invasive fibroblasts (top) with regards to the spatial distribution of F-actin (left) and hyaluronan (middle) and merged (right) columns. Arrows indicate extracellular hyaluronan trails. Scale bar: 20 μm.

To understand the role of hyaluronan in the cell adhesion of metastasising child fibroblasts, we analysed the spatial distribution of hyaluronan, focal adhesions and the actin microfilament system in isogenically matched normal and invasive child fibroblasts. In both invasive and control cells, hyaluronan showed mainly a cytoplasmic localisation. However, hyaluronan was also found in the extracellular environment, it localised where a cell has resided, leaving trails after migrating cells (Fig. 1). The invasive cells showed more often hyaluronan at the cell periphery, and the signal was increased, as compared to control (Fig. 1).

### Transformed and invasive child fibroblasts show increased assembly of nanometre clusters of Hyaluronan at the plasma membrane of the leading edge of migrating cells

Although there are strong links between hyaluronan and cell motility^33^, the spatial distribution of hyaluronan in cells in the leading edge of cells at the nanometer level is not known. We therefore characterised the nanoscale spatial distribution of hyaluronan at the lamellipodia of cells, and if this is altered in invasive cells, as compared to non-invasive control cells, using super-resolution STED microscopy. In line with previous observations, we observed increased filamentous actin and actin ruffles at the leading edge of invasive cells, as well as smaller and less distinct cell–matrix adhesions, as compared to normal control^12,34^.

**Figure 2:**
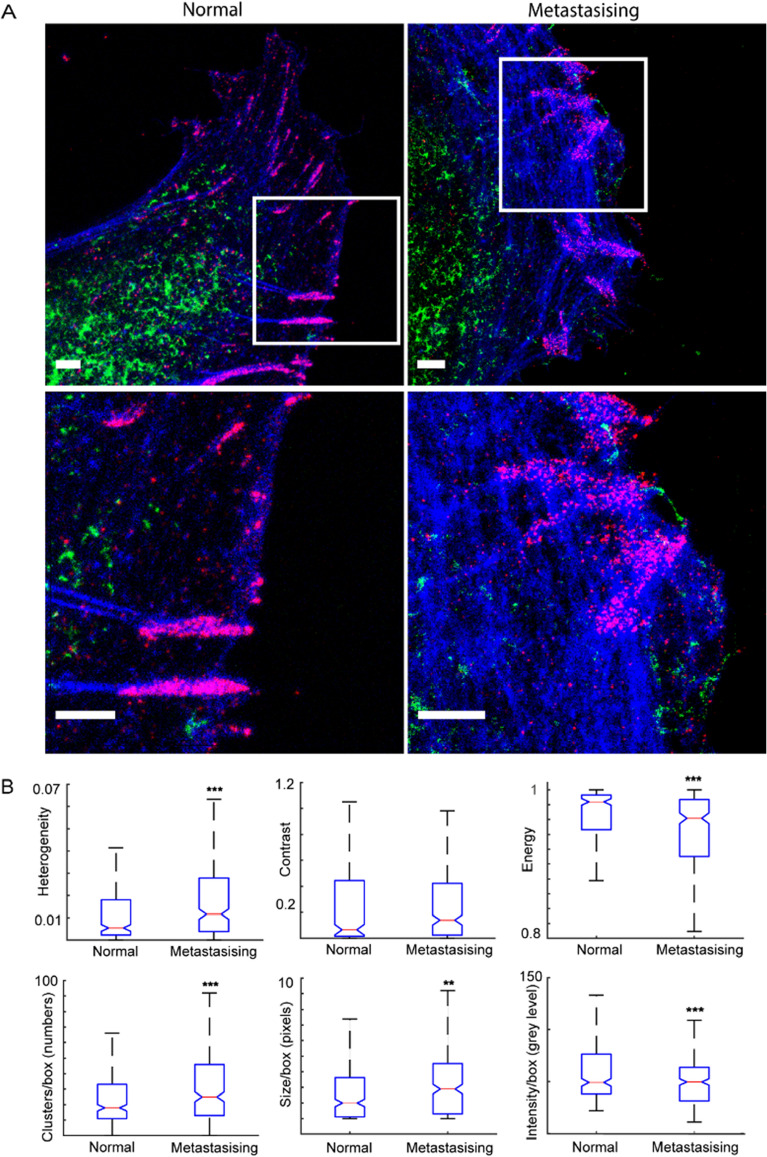
Nanoscale hyaluronan distribution in normal and invasive fibroblasts. (**A**) top panel, spatial distribution of hyaluronan (green), phosphotyrosines (red) and F-actin (blue) in control Bjhtert (left) and Bj-invasive (right) cells; lower panel, magnified boxed areas. (**B**) Notched box plots of hyaluronan features, thin red line showing median values. *** and ** indicates *p* < 0.001 and *p* < 0.01, respectively. Scale bars indicate 2 µm.

We observed that hyaluronan was localised in the same subcellular region in the leading front of the cells, but we could not detect a co-localisation to cell–matrix adhesions. Rather, hyaluronan was not observed within focal adhesions. However, the invasive cells showed hyaluronan at the very cell periphery, often in longer aggregates, which was not observed in control cells (Fig. 2A). To further identify differences between the distribution of hyaluronan in the front of invasive and control cells, the STED imaging was followed by computational image analysis, as described in the Material and Method section. For this, we first analysed the heterogeneity and the contrast of the signal in the images and observed that the heterogeneity of the pixels was higher and more widely distributed in the invasive cells, as compared to control, and that the typical contrast in the signal, in the invasive cells, was significantly higher compared to the control (Fig. 2B, Suppl. Fig. S6 and Suppl. Table S2).

We further observed that the signal energy, a measure of the homogeneity of the signal of the pixels, was lower in the invasive cells, as compared to control. Thereafter, we analysed to which degree hyaluronan was assembled in nanoscale clusters. We observed that the hyaluronan in the invasive cells was distributed in a higher number of clusters per cell surface area, and these clusters were 50% larger as compared to clusters in control cells. The average pixel intensity of these clusters did not significantly differ between control and invasive cells (Suppl. Fig S4). However, the signal of invasive cells showed lower pixel intensity per cluster compared to the control. Under the assumption that pixel intensity is proportionally related to the density of hyaluronan within the pixel boundary, these observations indicate that hyaluronan tends to be more assembled into larger clusters, that these clusters are closer to each other spatially and individual clusters are less densely packed with hyaluronan in invasive cells as compared to in non-invasive cells (Fig. 2A).

### Hyaluronan levels are increased at the periphery of Infantile fibrosarcoma tumours

**Figure 3:**
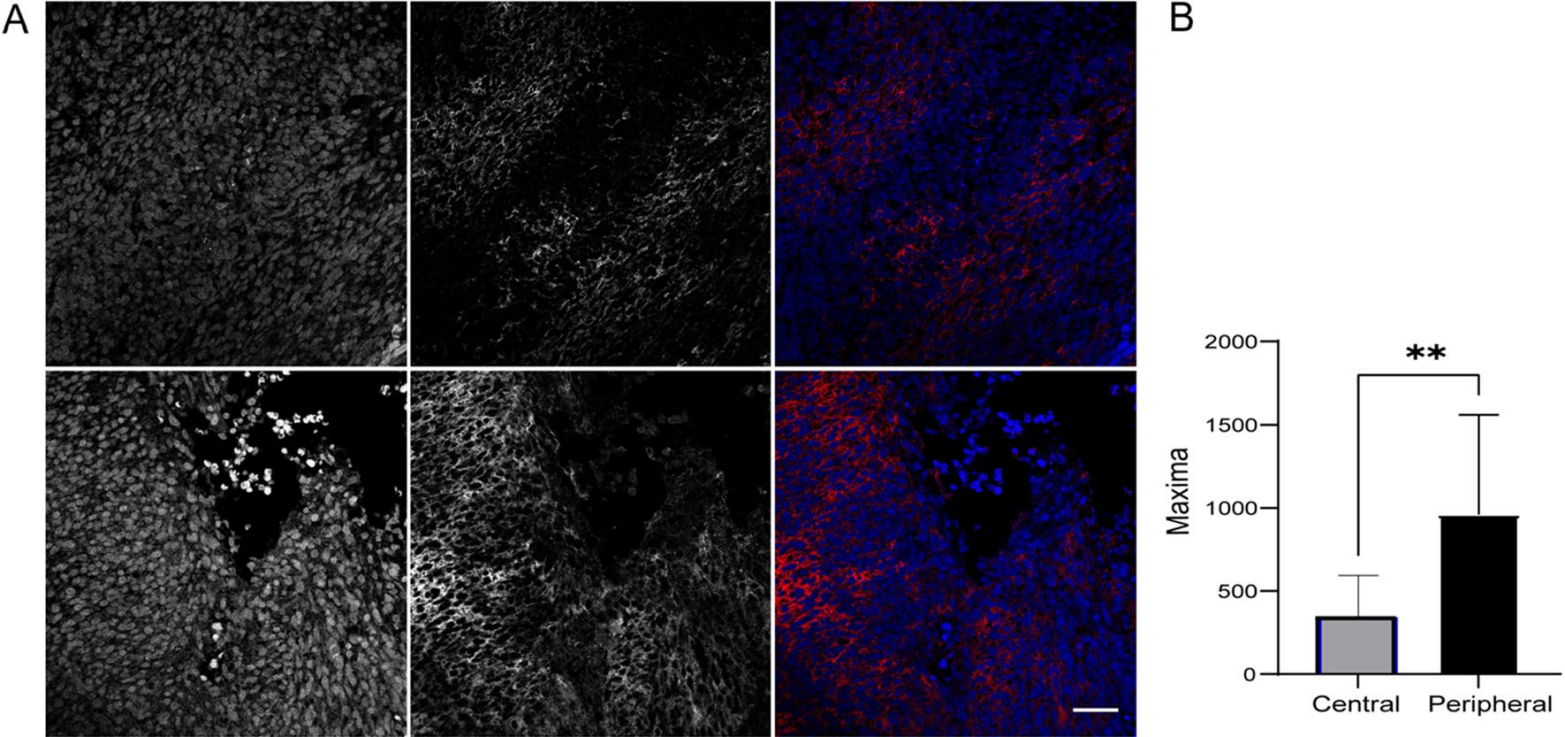
Hyaluronan distribution in Infantile fibrosarcoma. (**A**) Central (top), and peripheral (lower panel) areas of infantile fibrosarcoma, showing DAPI (left), hyaluronan (middle), and merged DAPI (blue) Hyaluronan (red) (right). Scale bar indicates 40 µm. (**B**) Hyaluronan levels at the peripheral (black) and central (grey) areas of infantile fibrosarcoma tumour sections. ** indicates p < 0.01.

We then wished to determine if hyaluronan can promote the invasion of child fibrosarcoma tumours into the surrounding tissue. For this, we compared the levels of hyaluronan in the central and peripheral regions of tumour sections from patients with infantile fibrosarcoma. The Paediatric oncology team considered the tumour to be aggressive, and to have a definite potential to spread prior to excising the tumour (oral communication). The levels of hyaluronan at the peripheral areas of the tissue appeared to be increased, as compared to the central regions of the tumour (Fig. 3 and Suppl. Fig. S5).

### Hyaluronan synthases interact with known regulators of cell motility

With the aim to identify candidate proteins that regulate or mediate the effect of hyaluronan on cell motility and infantile fibrosarcoma invasion, we performed a systematic literature review, and STRING analysis, as described in the Material and Method section. Thereby, we identified Hyaluronan synthases 1–3, Hyaluronidase 1–5 and their interactors, as shown in Supplementary figure S2. To clarify the signalling pathways involving these proteins, we analysed their known protein–protein interactions between these proteins. As expected, hyaluronan synthase- and hyaluronidase- interactors clustered to two separate groups of proteins (Suppl. Fig. S3). The highest confidence of interaction was observed between the hyaluronan receptor CD44 and hyaluronidases. To further clarify the molecular mechanism by which hyaluronan can control cell motility and cancer invasion, we analysed known functions of the identified interactors. In contrast to Hyaluronan synthase 1–3 that showed no strong interactions with any other proteins (Suppl. Fig. S2), Hyaluronidase 1–5 all showed strong interactions with the following proteins: GUSB, ARSB, IDUA, CD44, RHAMM, STAB2, CHP1, SLC9A, LYVE, IDS and GALNS (Suppl. Fig. S3). The analysis further showed interaction between CD44 and all five hyaluronidases.

### Transformed and invasive fibroblasts show increased gene expression of Hyaluronan synthase 2 and reduced expression of Hyaluronidase 2 and CD44

We then wished to identify novel hyaluronan-related molecular mechanisms by which child fibroblasts can gain the capacity to invade and metastasise. To this end, we compared the expression of the genes corresponding to the proteins identified as hyaluronan-interacting proteins above between the isogenetically matched invasive and control BJ child fibroblasts analysed shown in Figs. 1 and 2^12,34^. We observed that Hyaluronan synthase 2 and β-glucuronidase were upregulated in the invasive fibroblasts, with Hyaluronan synthase 2 showing the most statistically significant increase (Table 1).

**Table 1:** Up-regulated hyaluronan-related genes in invasive (Bj-invasive) relative to control child fibroblasts (Bjhtert).

Protein name	Protein abbreviation	Bj-invasive vs. Bjhtert (log_2_ change)	*p* value
β-Glucuronidase	GUSB	1.347	6.00 × 10^−3^
Hyaluronan synthase 2	HAS2	1.532	2.17 × 10^−38^

The gene expression corresponding to the following proteins was downregulated in the invasive cells, as compared to control: Hyaluronidase 2, CD44, Iduronate 2-Sulfatase, Galactosamine (N-Acetyl)-6-Sulfatase, Alpha-L-Iduronidase, CD44, Calcineurin Like EF-Hand Protein 1, Solute Carrier Family 9 Member A1. Of these, CD44 showed the most statistically significant down-regulation (Table 2). The Hyaluronan-related genes that did not show a statistical change in expression are shown in Supplementary table S1. The genes coding for the identified hyaluronan-related proteins Hyaluronidase 5/SPAM1, RHAMM, STAB2, KY, LYVE1 were not expressed in these cells^34^.

**Table 2:** Down-regulated hyaluronan-related gene expression in invasive (Bj-invasive) relative to normal control child fibroblasts (Bjhtert).

Protein name	Protein abbreviation	Bj-invasive vs. Bjhtert (log_2_ change)	*p* value
1 Duronate 2-Sulfatase	IDS	− 0.320	1.66 × 10^−4^
Galactosamine (N-Acetyl) 6-Sulfatase	GALNS	− 0.793	1.13 × 10^−6^
Alpha-L-Iduronidase	IDUA	− 2.257	4.56 × 10^−13^
CD44	CD44	− 1.306	6.73 × 10^−68^
Calcineurin Like EF-Hand Protein 1	CHP1	− 0.673	2.16 × 10^−8^
Solute Carrier Family 9 Member A1	SLC9A1	− 0.461	2.00 × 10^−3^
Hyaluronidase 2	HYAL2	− 0.389	1.10 × 10^−2^

### Expression of Hyaluronidases 3 and 5 in fibrosarcoma tumours is linked to increased mortality

To determine the importance of hyaluronidases on the progression of fibrosarcoma, we analysed survival of patients expressing the genes encoding for Hyaluronidase 3 and 5 using the Cancer Genome Atlas, as described in the Material and Method section. Patients with fibrosarcoma tumours expressing genes encoding for Hyaluronidase 3 or 5 showed a worse prognosis when compared to the overall patient cohort. The three-year survival rate for patients whose tumour expressed the genes corresponding to Hyaluronidase 3 and 5 was 89% and 36%, respectively. This should be compared to that of 93% for the overall patient cohort (Table 3). All hyaluronan synthases and hyaluronidases, with the exception of Hyaluronidase 1, were linked to reduced survival of patients diagnosed with fibrosarcoma (Suppl. Figure S7). As hyaluronidases are responsible for cleaving high molecular weight hyaluronan into low molecular weight variants, an adverse outcome on survival rates supports the hypothesis that low molecular weight hyaluronan promotes metastasis. Due to lack of bioinformatic data, Hyaluronidase 5 is absent from this analysis. Taken together, these results indicate that expression of hyaluronidases can contribute to the progression and invasion of fibrosarcoma tumours.

**Table 3:** Mortality of child fibrosarcoma expressing hyaluronidases and the overall patient cohort^29^.

Protein name	Gene name	3-Year patient survival rate (%)
Hyaluronidase 3	HYAL3	89
Hyaluronidase 5	HYAL5	36
	Overall	93

## Discussion

The nanoscale spatial organisation of hyaluronan at the leading edge of cells, and how hyaluronan regulates the formation and function of focal complexes and focal adhesions remain to a large extent unknown. We observed, for the first time, that the leading front of invasive child fibroblasts show a more clustered organisation of hyaluronan at the nanometer level, as compared to normal control fibroblasts. This highlights the possibility that the nanoscale clustering of cleaved, low molecular weight variants of hyaluronan organise into assemblies that induce the initial steps of cell adhesion, focal contacts, cell force and cell motility. We did not observe hyaluronan within focal adhesions. Taken together with recent observations that the local degradation of surface bound hyaluronan at mature focal adhesions is required for cell migration^35^, this suggests that while high molecular weight variants of hyaluronan at focal adhesions suppress cell migration, low molecular hyaluronan at the leading edge promotes the formation of focal complexes and cell migration. Recent findings have shown that CD44 can function as pickets, fixing the cortical actin to the membrane and to hyaluronan, which limits the mobility of receptors at the cell surface. It is possible this is due to that CD44 creates a physical barrier that prevent lateral diffusion of receptors^36^. Taken together with our observations, this suggests that the spatial distribution of hyaluronan, at the nanometer level, governs the biochemical signalling at the cell surface, and thereby cytoskeletal dynamics, cell morphology and function.

Hyaluronan has been shown to regulate the motility of other types of cancers than fibrosarcoma, in particular breast carcinomas^37–40^. Low molecular weight hyaluronan promotes inflammation and the formation of the tumour-promoting stroma, while the total amount of hyaluronan correlates to lymph node metastasis and predicts poor overall survival^41,42^. Our observation that gene expression of Hyaluronan synthase 2 and 3 and Hyaluronidase 2, 3 and 5 in fibrosarcoma patients is linked to reduced patient survival is consistent with the observation that Hyaluronan synthase 1 but not 2 suppresses the motility of adult fibrosarcoma cells, by synthesising high molecular weight variants of hyaluronan, which increase cell adhesion of fibrosarcoma cells^37^. Our observations are also in line with the finding that hyaluronidase-mediated degradation of hyaluronan induces cell motility^35^. Together, this is in line with the possibility that the binding of extracellular, not surface bound, low molecular weight hyaluronan to the receptors RHAMM and CD44 induces the formation of cell adhesion, cell motility and invasion of fibrosarcoma.

We observed that the invasive child fibroblasts showed decreased expression of CD44. This contrasts with earlier findings, which report that different types of invasive fibrosarcoma cells show increased expression of CD44^37^. These conflicting observations could be explained if paediatric fibroblasts and Infantile fibrosarcoma is less dependent on the levels of CD44 than and adult fibroblasts and fibrosarcoma. In line with this possibility, decreased expression of CD44 has been shown in cells to not impact on the ability of cells to adhere^6^. In addition, child fibroblasts have an increased capacity to migrate, and different gene expression profiles, as compared to adult fibroblasts^43^. Hence, it is possible that the role of hyaluronan, and the receptors to which it binds are slightly different in paediatric as compared to adult fibrosarcoma.

Our observation that invasive and invasive child fibroblasts show increased gene expression of Hyaluronan synthase 2, but not 1, is consistent with earlier findings suggesting that Hyaluronan synthase 2 is the main hyaluronan synthase in fibroblasts^41^. It is also in line with the previous observation that only Hyaluronan synthase 1, but not 2 suppresses fibrosarcoma cell motility. The importance of Hyaluronan 2 is further highlighted by the observation that loss of Hyaluronan synthase 1 and 3 in fibroblasts results in a compensatory upregulation of Hyaluronan 2 activity and increased levels of hyaluronan^41^. Hyaluronan 2 is further required and sufficient for the invasion of normal adult fibroblasts^37^. It is important to put our gene expression data in the context of this presented functional data, because Gene expression studies are limited to proteins that have steady state concentrations of mRNA. This excludes proteins mainly regulated at the post-translational level, such as the protein RHAMM^44^. Taken together with the previous observations indicated above, our findings, the possibility that Hyaluronan synthase 2 can control the spread of Infantile fibrosarcoma.

To our knowledge, our study presents the first evidence of a role of hyaluronan in invasive child fibroblasts and Infantile fibrosarcoma. It is also the first to show the nanoscale spatial distribution of hyaluronan, in relation to focal adhesions and the actin cytoskeleton. Our data suggests that the nanoscale clustering of hyaluronan at the leading edge of fibroblasts can promote cell motility, and that increased levels of hyaluronan, Hyaluronan Synthase 2, Hyaluronidase 3 and 5 can contribute to the spread and invasion of Infantile fibrosarcoma.

## Conclusion

In summary, our data are in line with the hypothesis that increased levels and a specific and distinct nanoscale spatial distribution of hyaluronan in the leading edges of invasive child fibroblasts, and increased levels at the front of Infantile fibrosarcoma tumours contribute to tumour invasiveness and the outcome of infantile fibrosarcoma patients.

## Supplementary Information

Supplementary Information.

## Data Availability

The data supporting the findings of this study are available from the corresponding author upon reasonable request.
