# Supplementary material for: Hyaluronan nanoscale clustering and Hyaluronan synthase 2 expression are linked to the invasion of child fibroblasts and infantile fibrosarcoma in vitro and in vivo
Source: Sci Rep. 2022 Nov 18;12:19835. doi: 10.1038/s41598-022-21952-4 (PMC9674583; doi:10.1038/s41598-022-21952-4)
Supplement: Supplementary file 1 — Supplementary Information. [file 41598_2022_21952_MOESM1_ESM.docx]

**Supplementary figures, tables and equations**


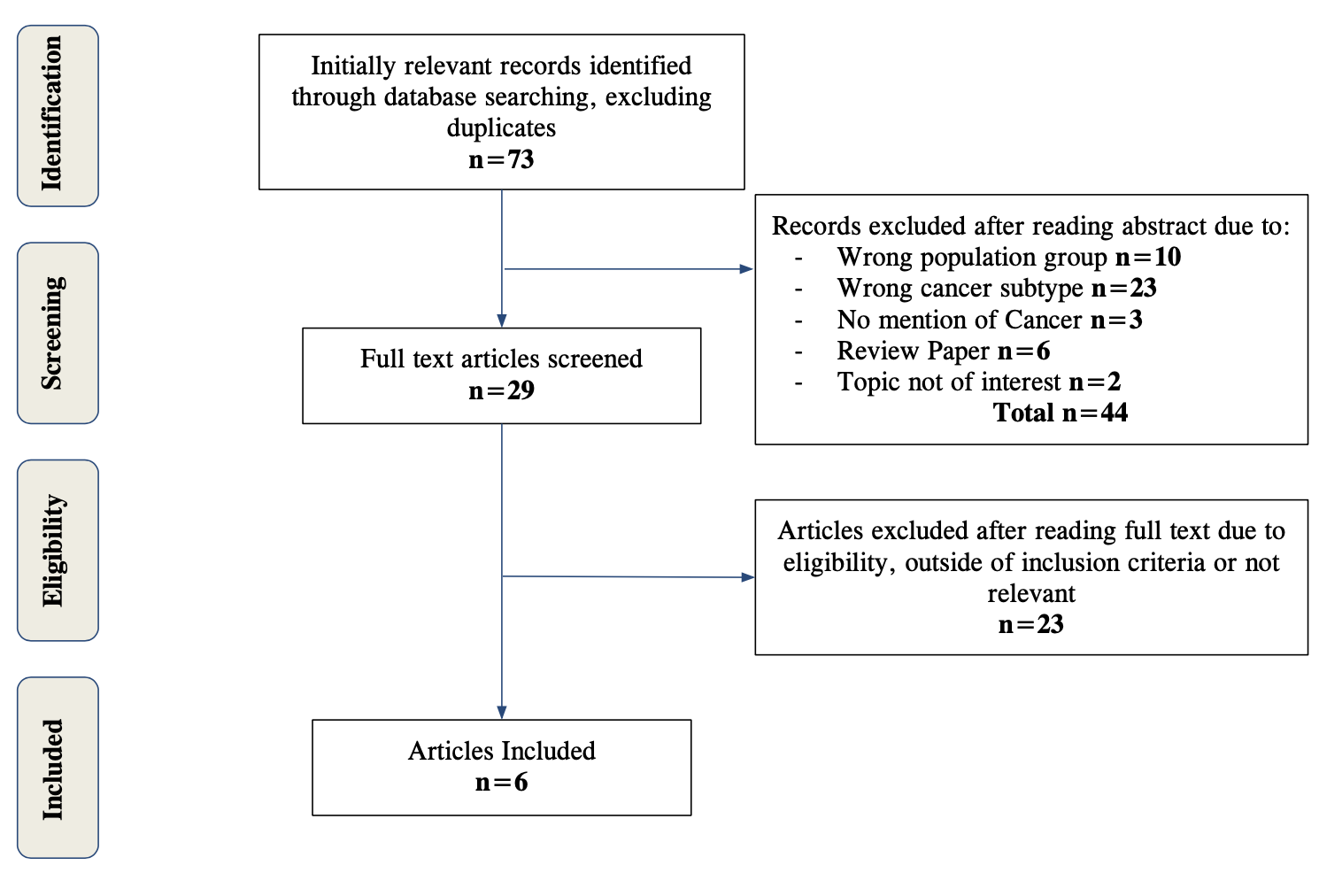


**Figure S1: Systematic literature review process.** Flow chart showing the approach used to identify research articles describing the role of hyaluronan of pediatric fibrosarcoma.


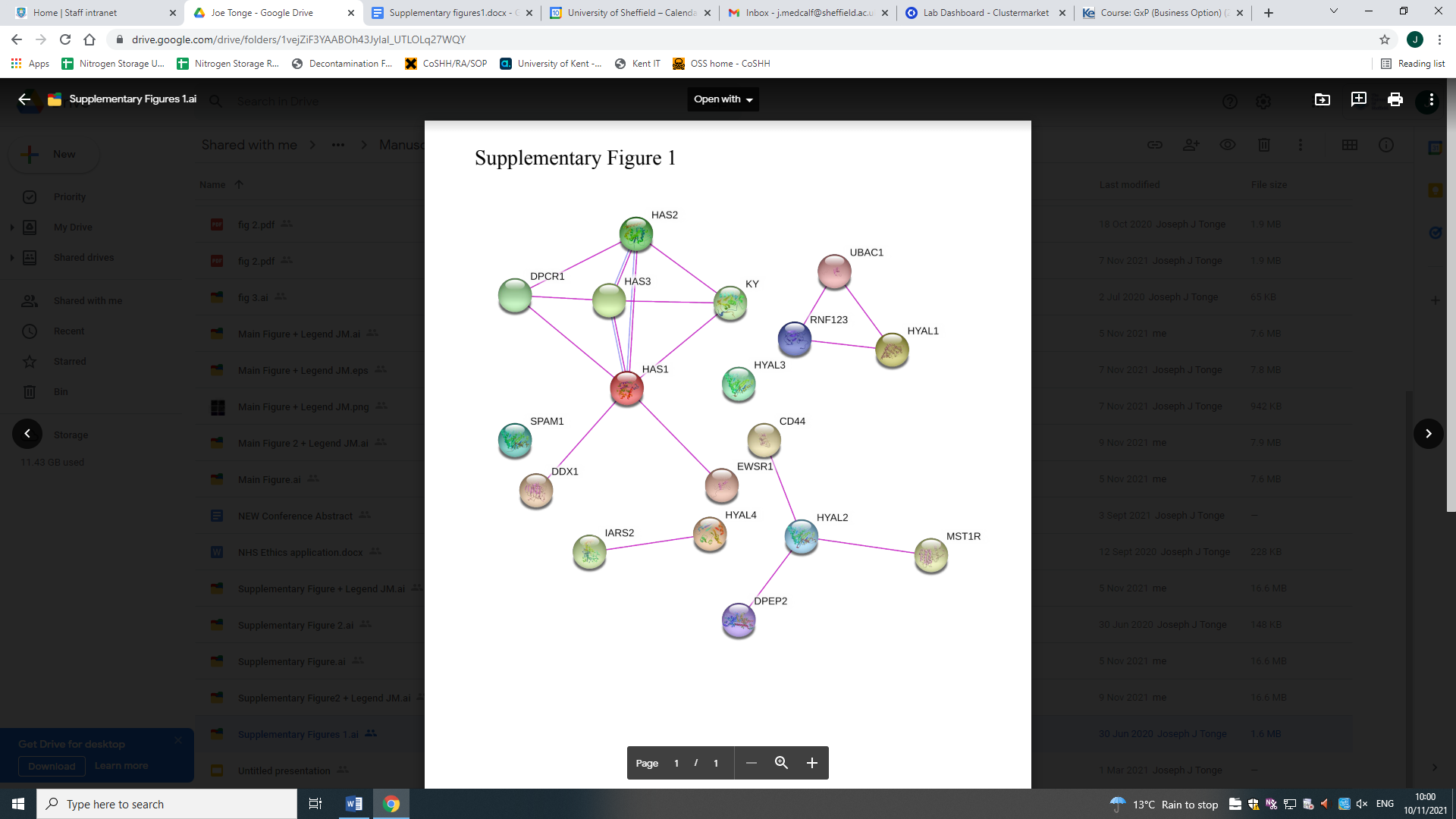


**Figure S2: STRING analysis of the protein interactions of hyaluronan synthases and hyaluronidases.** Lines indicate experimentally shown (magenta) or computationally predicted (turquoise) interactions, as well as protein homology (light blue). Circular nodes show the indicated proteins.

.


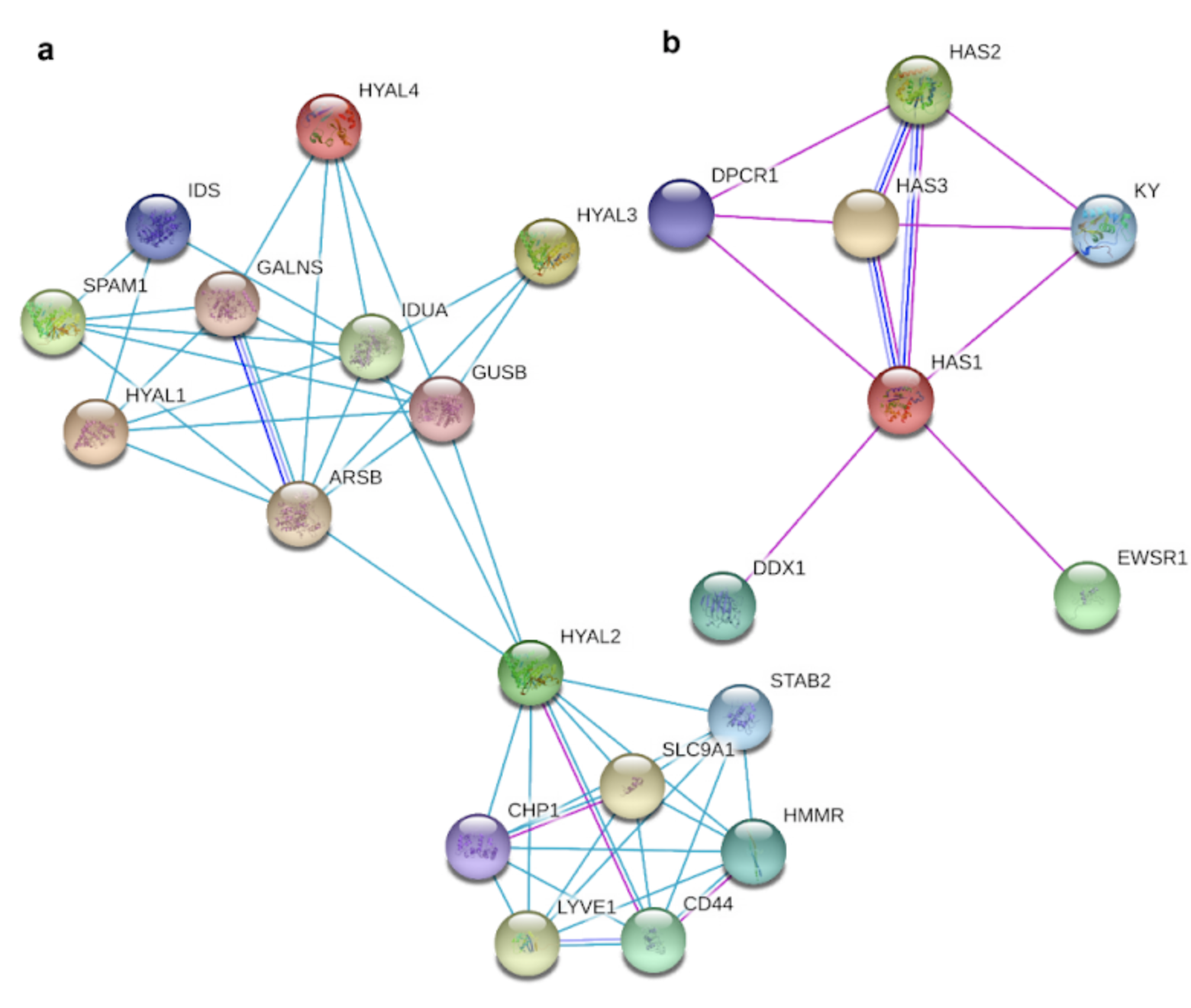


**Figure S3. STRING analysis of interactions between hyaluronan-related proteins identified in the systematic literature review.** The interactors of hyaluronidases (a), hyaluronan synthases (b) are shown, with lines indicate experimentally shown (magenta) or computationally predicted (turquoise) interactions, gene co-occurrence (dark blue), protein homology (light blue). Circular nodes show the indicated proteins.


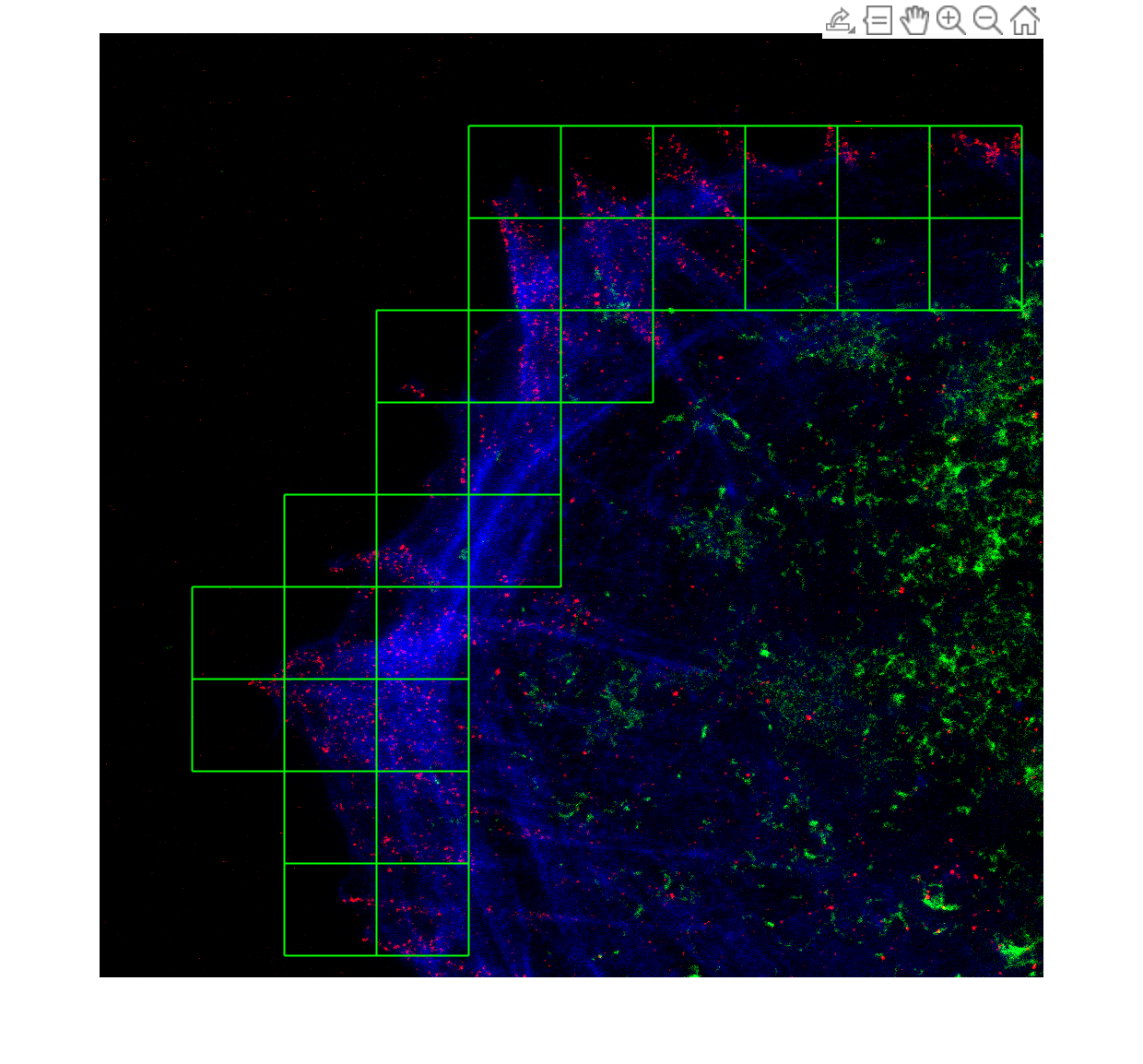


**Figure S4: Example of image segmentation defining the peripheral regions of cells into grid blocks of constant block size prior to computational analysis.** Image showing grid blocks of 2.52 x 2.52 micrometers (100 x 100 pixels) (green squares) in a 25.83 x 25.83 micrometer super-resolution STED image showing hyaluronan (green), phosphotyrosines (red) and F-actin (blue).

**Figure S5: Hyaluronan levels and distribution in Infantile fibrosarcoma.** Central and peripheral areas of transverse Infantile fibrosarcoma sections, as indicated, showing DAPI (left panel), hyaluronan (middle panel), and merged images (right panel). Negative control for staining is shown as indicated. Scale bar indicates 20µm.
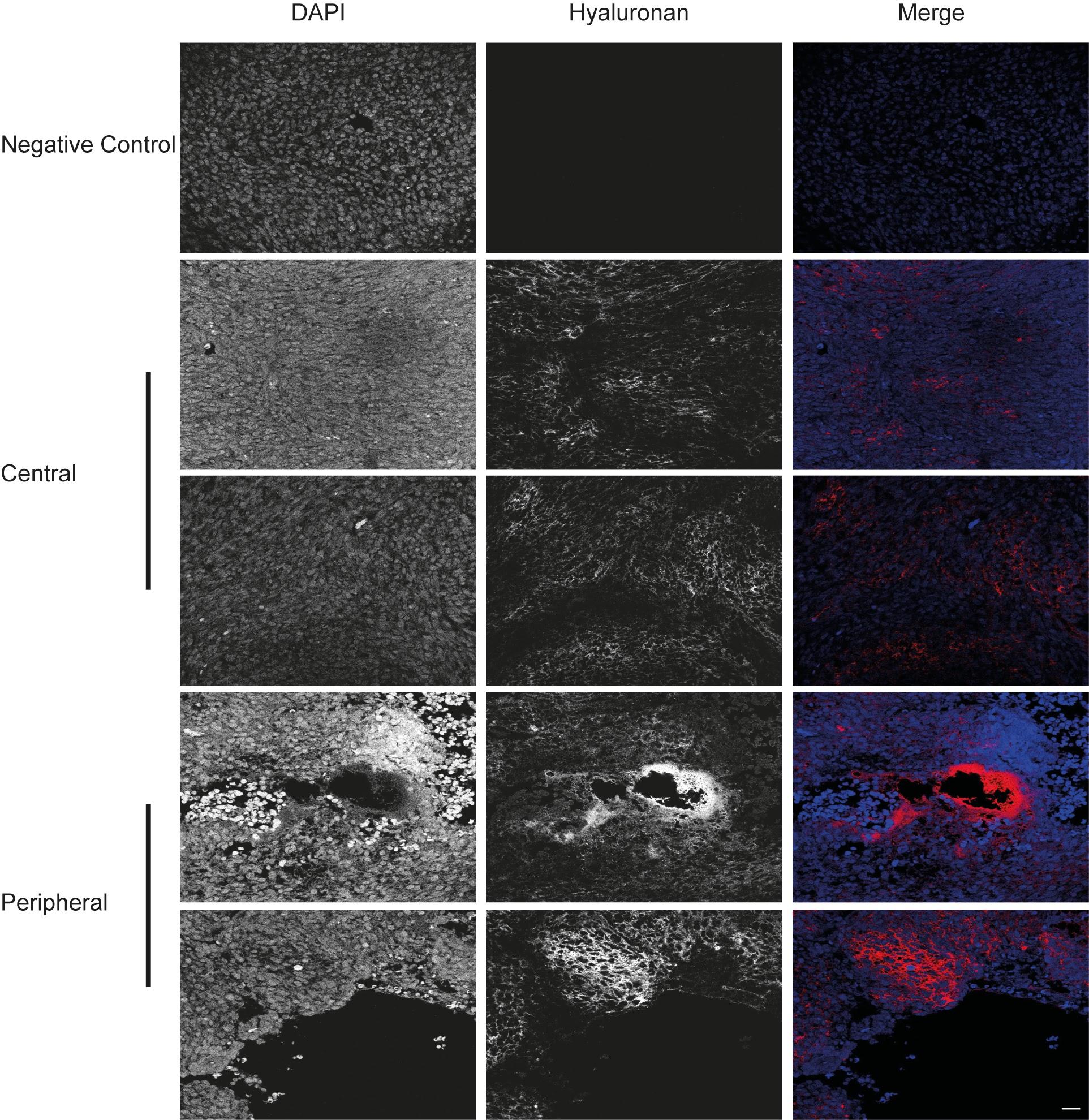


**
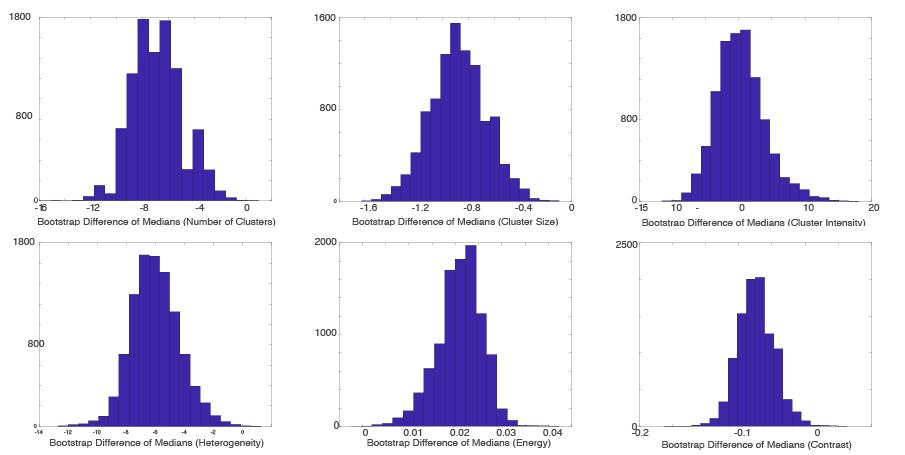
**

**Figure S6: Bootstrap distributions (10,000 samples) on the difference of medians for the nanoscale hyaluronan spatial distribution measures.** top panel, left to right; Average number of clusters per block, average cluster size per block, and average intensity per block. Lower panel; left to right; Heterogeneity, Energy, and contrast estimated within each block.


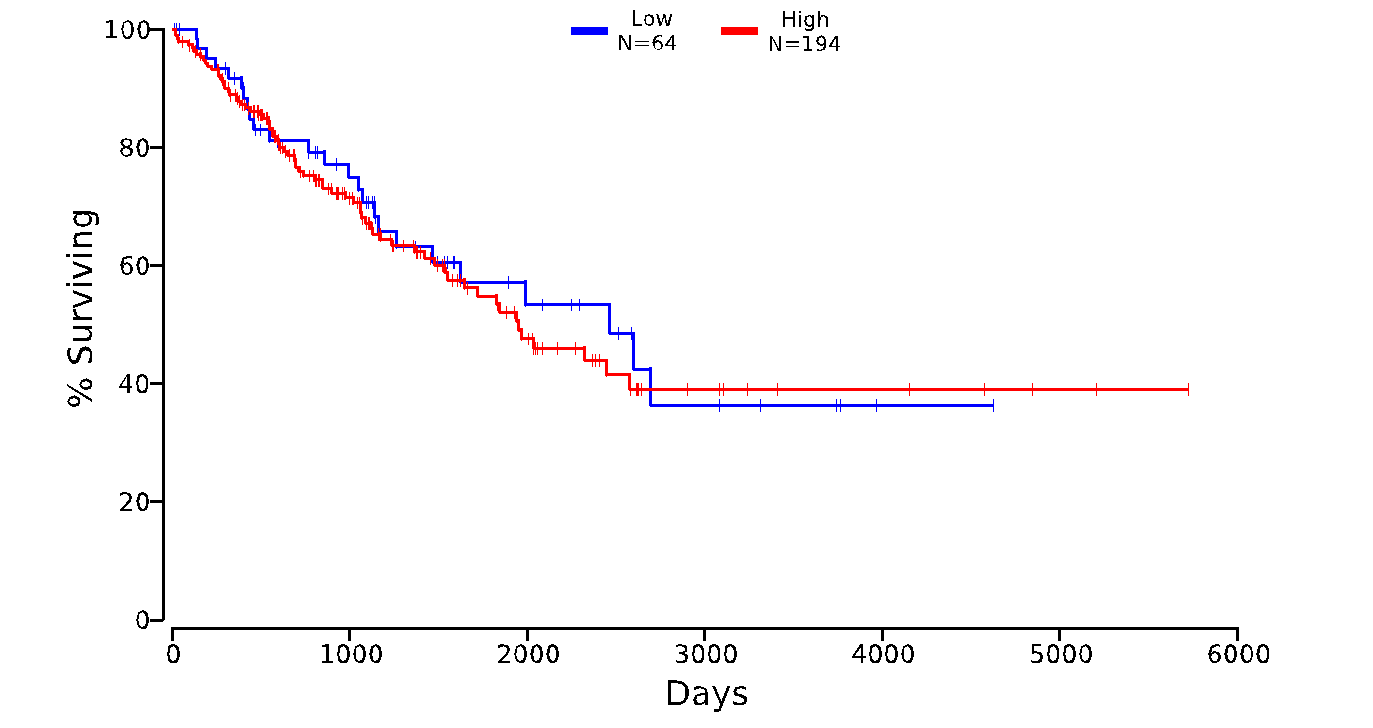

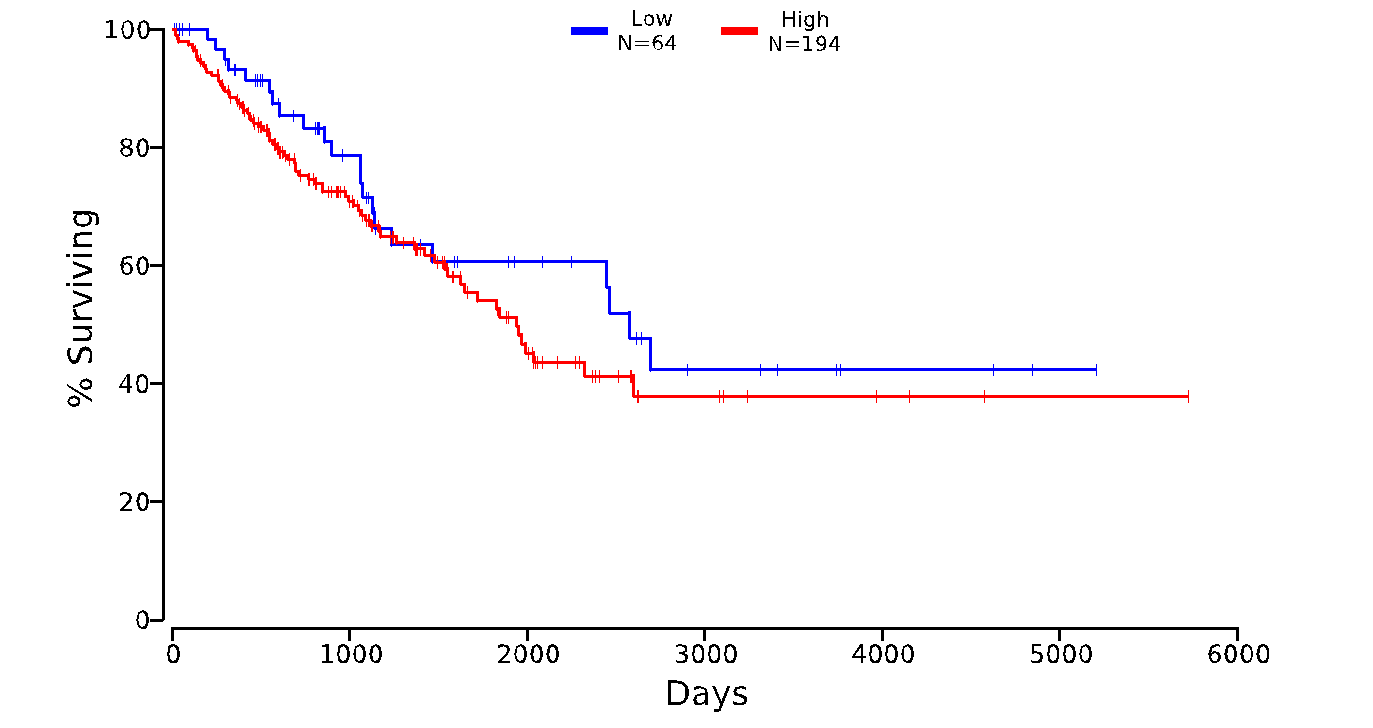


**a**

**b**

**c**


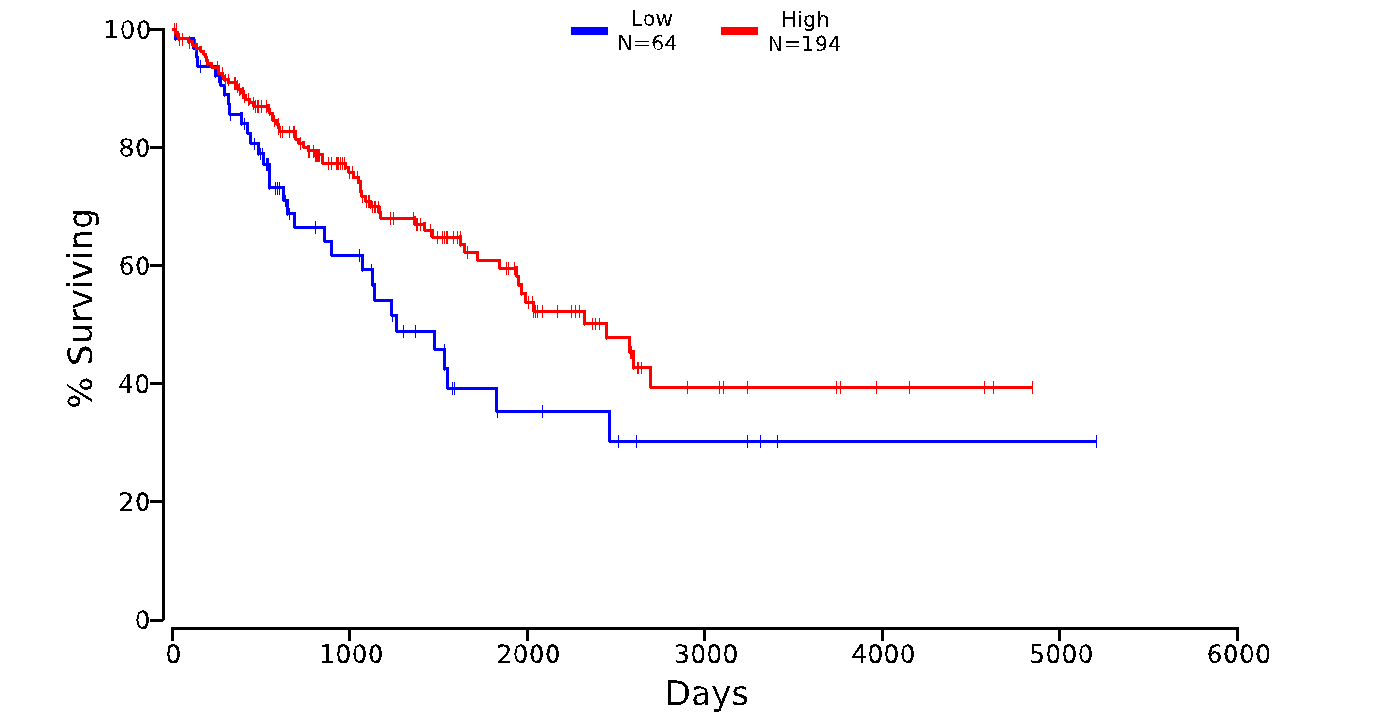

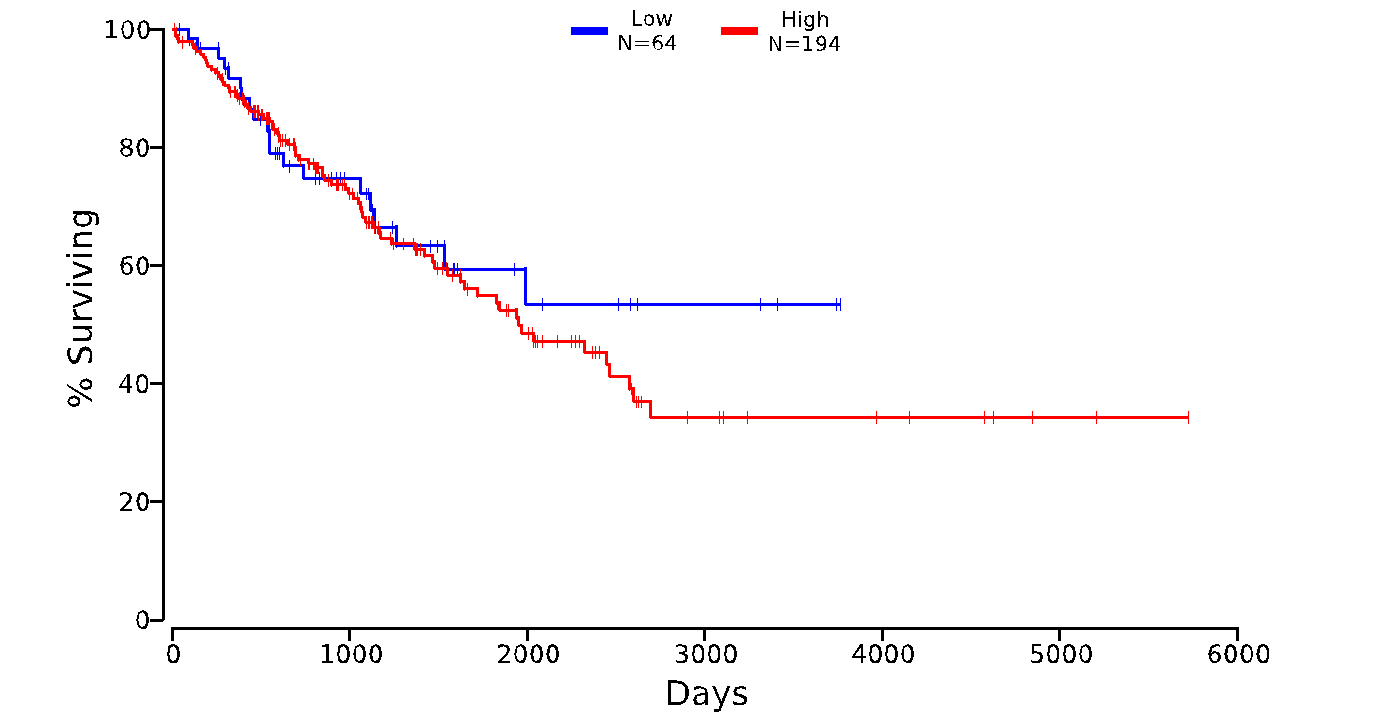


**d**

**f**

**e**


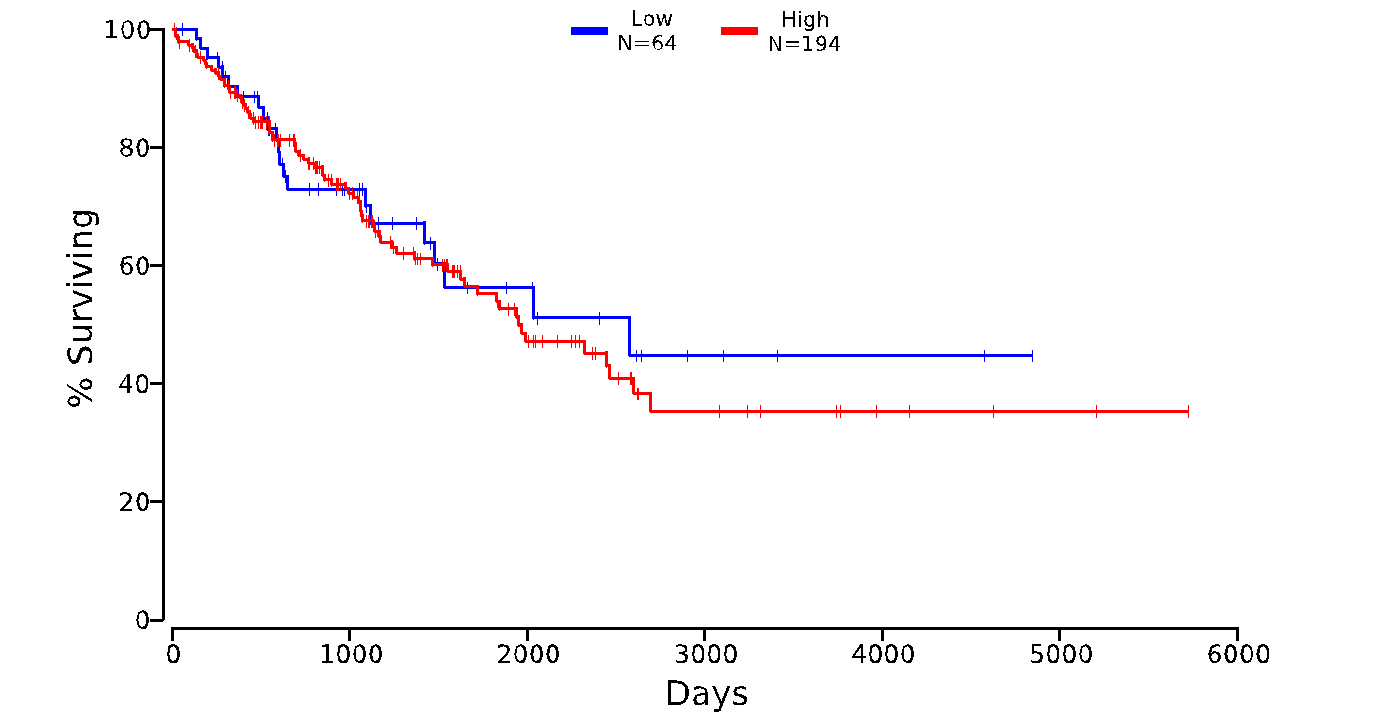

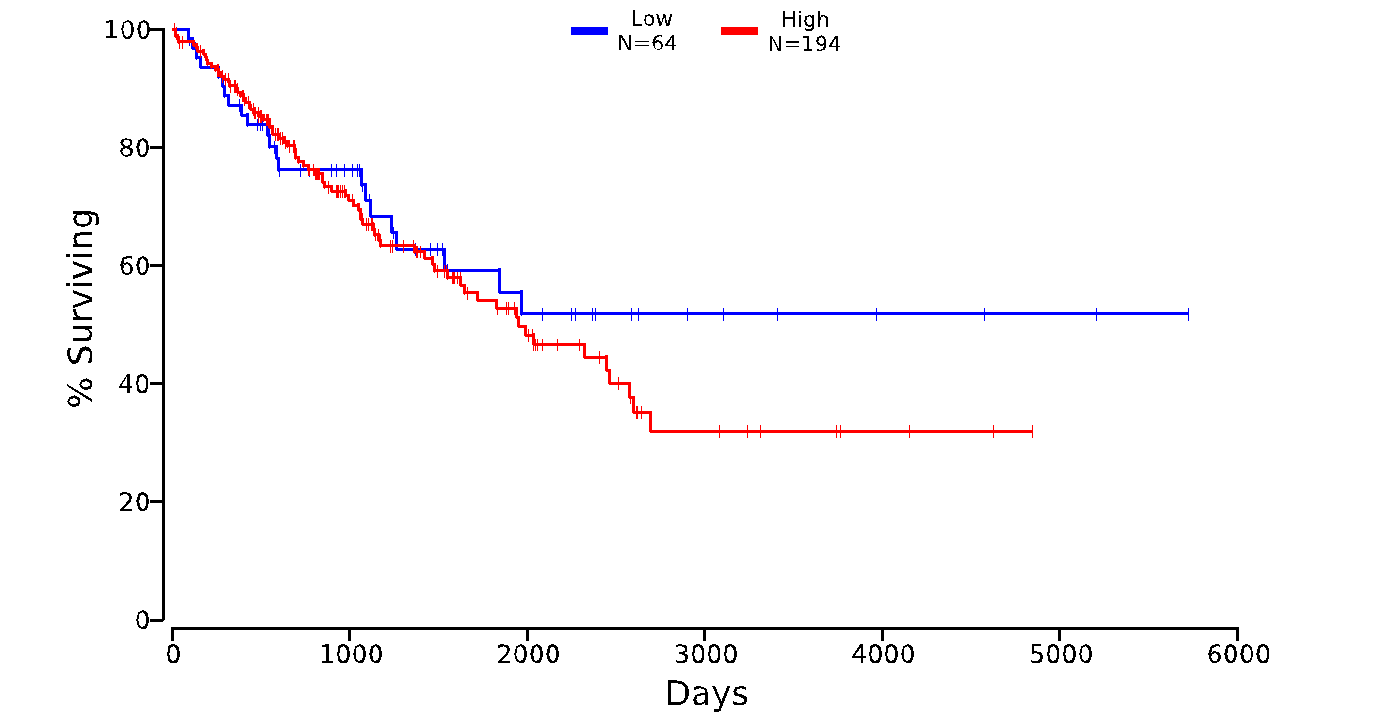


**Figure S7.** **Kaplan-Meier Survival curves for patients diagnosed with fibrosarcoma tumours**. Survival with low (blue) and high (red) expression of HAS1 (**a**), HAS2 (**b**), HAS3 (**c**), HYAL1 (**d**), HYAL2 (**e**), HYAL3 (**f**) genes.

**Supplementary tables**

**Table S1. Differential gene expression in metastasising, relative to normal child fibroblasts**

| **Protein Name** | **Protein Abbreviation** | **Bj-metastasising *vs* Bjhtert (log_2_ change)** | **p-value** |
| --- | --- | --- | --- |
| **Hyaluronan Synthase 1** | **HAS1** | **-1.721** | **0.517** |
| **Hyaluronidase 1** | **HYAL1** | **0.196** | **0.873** |
| **Hyaluronidase 3**    **Hyaluronidase 4** | **HYAL3**  **HYAL4** | **-0.012**    **0.966** | **0.973**    **0.846** |
| **Hyaluronidase 5** | **HYAL5** | **1.295** | **0.795** |
| **Arylsulfatase B** | **ARSB** | **-0.089** | **0.456** |
|  |  |  |  |

**Table S2. Mann-Whitney U p-values (double sided) and bootstrap derived z-scores between group medians for morphological and texture-based measures**

| **Measure** | **p-value** | **z-score** |
| --- | --- | --- |
| **Number of clusters per block** | **p<0.001** | **3.5** |
| **Average cluster size per block** | **p<0.01** | **4.0** |
| **Average cluster intensity per block** | **p<0.001** | **0.1** |
| **Heterogeneity** | **p<0.001** | **3.7** |
| **Energy** | **p<0.001** | **4.6** |
| **Contrast** | **p=0.162** | **2.6** |

**Supplementary equations**

The following equations define the calculations for energy, contrast and heterogeneity where $P(i,j)$ is the normalised GLCM matrix for an image patch.

$$energy=\sum_{i,j} {P(i,j)}^{2}$$

$$contrast=\sum_{i,j} \left( i-j \right)^{2}P(i,j)$$

$heterogeneity=1-H$ where $H=\sum_{i,j} \frac{1}{1+\left( i-j \right)^{2}}P(i,j)$
